# The Structure of Cross‐β Tapes and Tubes Formed by an Octapeptide, αSβ1†

**DOI:** 10.1002/anie.201207699

**Published:** 2013-01-10

**Authors:** Kyle L. Morris, Shahin Zibaee, Lin Chen, Michel Goedert, Pawel Sikorski, Louise C. Serpell

**Affiliations:** ^1^ School of Life Sciences, University of Sussex, Falmer, Brighton, East Sussex, BN1 9QG (UK); ^2^ MRC Laboratory of Molecular Biology, Cambridge, CB2 0QH (UK); ^3^ Department of Physics, Norwegian University of Science and Technology, Trondheim (Norway); ^4^ Centre for Materials Discovery, University of Liverpool, Crown Street, Liverpool, L69 7ZD (UK)

**Keywords:** helical structure, nanotubes, peptides, self‐assembly, X‐ray diffraction

## Abstract

**Elaborate morphology**: The αSβ1 peptide, a fragment of α‐synuclein, assembles into flat tapes consisting of a peptide bilayer, which can be modeled based on the cross‐β structure found in amyloid proteins. The tapes are stabilized by hydrogen bonding, whilst the amphiphilic nature of the peptide results in the thin bilayer structure. To further stabilize the structure, these tapes may twist to form helical tapes, which subsequently close into nanotubes.

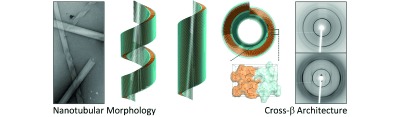

A number of short peptides and larger proteins are able to self‐assemble to form cross‐β amyloid‐like fibrils.^1^ These β‐sheet peptides have been increasingly explored as potentially useful bionanomaterials due to their propensity to spontaneously assemble to form large complex structures from simple monomers.^2^ An eight‐residue fragment of the amyloidogenic Parkinson’s disease related peptide α‐synuclein,^3^ named αSβ1, assembles to form a helical nanostructure. Here we report the supramolecular and molecular architecture of these helical nanotubes. The supramolecular structure consists of two peptide molecules forming an amphiphilic bilayer that extends into a tape, which subsequently adopts a helical arrangement that closes to form the nanotube morphology. The structural model is proposed based on interpretation of fiber diffraction data from nanotube samples with two different textures. Analysis reveals a cross‐β structure within the tapes, which go on to form an elaborate cross‐β nanotubular architecture.

X‐ray fiber diffraction has been extensively used to reveal that many proteins and peptides are able to assemble to form a cross‐β structure in which β‐strands run perpendicular to the fiber axis and associate to form long‐range hydrogen‐bonded β‐sheets.1c, 4 The inherent strength of this arrangement is underlined by the similarity to the architecture of cross‐β silks.4a, 5 The non‐covalent self‐assembly of peptide monomers also underpins the spontaneous formation of elaborate fibrillar structures.^6^

α‐Synuclein (α‐syn) is a 140‐residue peptide that assembles to form amyloid‐like fibrils that are deposited in Lewy bodies in Parkinson’s disease.^3^ α‐Syn fibrils assembled in vitro have been confirmed to have a β‐sheet‐rich structure consistent with the amyloid cross‐β architecture.^7^ Solid‐state NMR studies on the cross‐β amyloid core of recombinant human α‐syn fibrils have indicated sets of β‐strands at discrete positions between 30–110.^8^ We have explored the assembly potential and structure of a segment of α‐syn corresponding to positions 37–44 (NH_2_‐VLYVGSKT‐COOH) herein referred to αSβ1. This particular sequence is of interest because at high concentrations the peptide forms nanotubular cross‐β assemblies. As shown in Figure 1 a, αSβ1 was observed to form helical nanostructures when assembled in water at high concentration. Negative stain transmission electron microscopy revealed nanotubular structures that appear to be composed of helical tapes (Figure 1 and Figure S1 in the Supporting Information), similar in appearance to those described by Shao et al.6b and Adamcik et al.^9^ We have studied mature αSβ1 structures and the morphologies we observe are comparable to those observed between 24 h and 28 days for the CapFF peptide reported by Adamcik et al.^9^ The αSβ1 structures vary in width from ca. 240 (i) to 335 nm (ii) where the narrower structure extends to reveal a helical tape unravelling from a tube‐like structure (Figure 1). The helical tape has a width of ca. 360 nm (iii), suggesting that the wider structures (ii) may in fact represent flat tapes that have entirely unravelled from the helical nanotubular structures. Helical tapes were observed to split into two (iv) before re‐joining to form the original helical tape structure, indicative of the non‐covalent stabilization of the tapes perpendicular to their long axis. This is also evident in the considerable variation in tape width that was also observed, as shown in Figure 1 b. The assembly of αSβ1 is reminiscent of other peptide nanotube formers but over larger dimensions than those currently reported for linear peptides that form tube diameters ranging from 10–104 nm.6a, 9, 10

**Figure 1:**
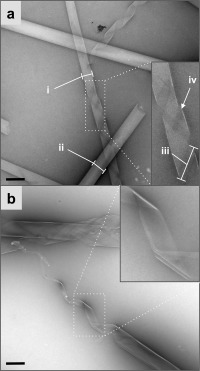
Negative stain transmission electron microscopy showing the nanotubular morphology of the self‐assembled αSβ1 peptide. a) The nanotubes have a diameter of ca. 240 nm (i) consisting of tapes unravelled from tubes with a width of ca. 335 nm (ii) to 360 nm (iii) which are found to occasionally laterally split (iv). b) Other representative assemblies reveal unravelling of the nanotube structure into helical ribbons of variable width. The scale bar represents 500 nm.

Fourier transform IR spectroscopy (FTIR) revealed that the αSβ1 peptide adopts a β‐sheet structure within the assemblies (Figure S2). Thus, the strongest set of non‐covalent interactions in the αSβ1 assemblies arises from cooperative β‐sheet backbone hydrogen bonding and so the high persistence length of the tapes suggests these interactions are aligned to the tape long axis. To probe the β‐sheet arrangement in the tapes that form the observed nanotubes, X‐ray fiber diffraction (XRFD) patterns were obtained from fibrous and film‐textured alignments of self‐assembled αSβ1 (Figure S3 and Table S1). Nanotubes and tapes are aligned with their long axes parallel to the macroscopic fiber axis in the fibrous texture and aligned parallel to the film plane in the film texture.

The diffraction signals expected from cross‐β structures were observed at 4.7–4.8 Å and 9.8 Å but with axial alignments different to traditional cross‐β amyloids, indicating a novel arrangement within the αSβ1 assemblies. The texture of the fibrous alignment is complex and we hypothesize that the patterns are complicated by contributions from both nanotubes and tapes, however they exhibit azimuthal reflection angles that are consistent with the helical alignment of the 4.8 and 9.8 Å periodicities (Figure S4a). The texture of the film‐textured alignment is simpler since flattened nanotubes will contribute to diffraction in the same way as flat tapes. Interestingly however, we observe azimuthal reflection angles consistent with helices (Figure S4b), similar to observations on other nanotubular assemblies.10b, 13 Inspection of the film‐textured XRFD pattern finds that the 4.8 and 9.8 Å reflections are aligned to the equator (Figure S3b) indicating their corresponding structural periodicities are both aligned parallel to the film plane. Of particular interest, a 29 Å reflection was observed to have meridional alignment indicating that the structural periodicity it arises from is aligned perpendicular to the film plane and thus perpendicular to the αSβ1 tape long axis. The observed periodicity of 29 Å correlates well with the length of the αSβ1 peptide in a β‐strand conformation of 28 Å (8 residues×3.5 Å). Therefore, we deduce that the long axis of the αSβ1 molecule is arranged perpendicular to the αSβ1 tape long axis (Figure S5). This is corroborated by the equatorial alignment of this reflection in the fiber‐textured alignment indicating the αSβ1 molecules are aligned perpendicular to the nanotube long axis. From these observations, the molecular orientation within the assemblies can be described, as shown in Figure 2, where the long axis of the αSβ1 peptide is aligned perpendicular to tape long axis and width. The nanotube supramolecular structure is consistent with the XRFD data and is further consistent with the amphiphilic nature of the molecule (Figure 2 d), whereby two αSβ1 peptides stack end‐on‐end within the wall to make a bilayer of a thickness of ca. 56 Å (16 residues×3.5 Å).

**Figure 2:**
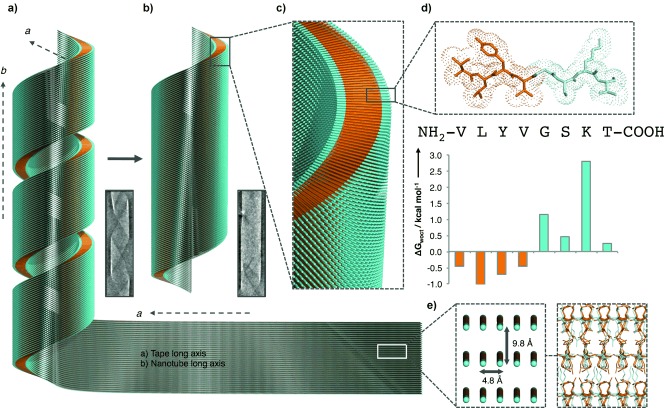
The proposed model of the arrangement of αSβ1 peptides that creates a nanotubular morphology. a) Helical tapes form and close into b) mature tubes. c) The peptides are arranged out‐of‐plane with respect to the tube wall creating an amphiphilic bilayer stabilized by d) the amphiphilic nature of the αSβ1 peptide. e) The orientation of the αSβ1 strands are shown in the context of the tape then leading to the nanotubes. The single peptides are represented as lines with hydrophobicity and hydrophilicity shown as orange and cyan, respectively. The hydrophobicity of the αSβ1 sequence is shown according to the White and Wimley scale.^11^ Graphics generated in Pymol.^12^

The reflections measured from the film‐textured XRFD pattern were found to index to an orthorhombic unit cell of the dimensions *a*=9.50; *b*=19.92; *c*=27.97 Å; *α*=*β*=*γ*=90° (Figure S6 and Table S2). These unit cell dimensions are consistent with the typical interatomic separations observed for the packing of short amyloid‐like peptide molecules in the β‐strand conformation. The 4.8 Å reflection is consistent with a hydrogen bonding separation of β‐strands (half of the *a* dimension) and the 9.8 Å arises from spacing between β‐sheets (half of the *b* dimension). The determined unit cell was found to accommodate four peptide molecules corresponding to half the amphiphilic bilayer of the nanotube wall (*c*=27.97 Å). The molecular architecture of the αSβ1 peptide within this cell was explored through iterative model building of both parallel and antiparallel models. Calculated X‐ray fiber diffraction patterns were compared to experimental X‐ray data until the backbone architecture best reproduced the experimental X‐ray fiber diffraction. The model structure was minimized to orient side chains and the final model constructed as shown in Figure 3. The β‐sheet arrangement was concluded to be parallel to maintain the amphiphilicity of the nanotube wall and this model was found to be consistent with diffraction data comparisons.

**Figure 3:**
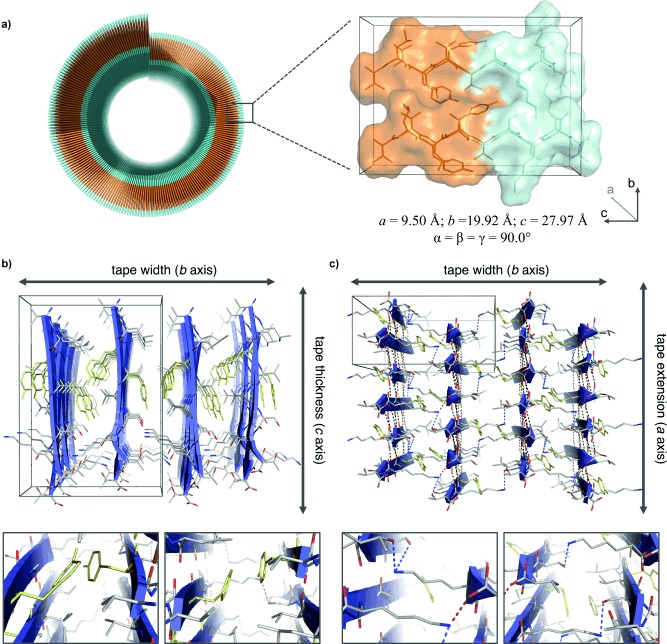
The molecular architecture of αSβ1 peptide in the context of the helical tape that constitutes the amphiphilic bilayer nanotube wall (colored for hydrophobic, orange; hydrophilic, cyan). a) The length of the peptide determines the tape thickness. b) The tape width is stabilized by the interdigitation of side chains and intersheet Tyr interactions as highlighted in yellow. c) The tape then extends through interstrand amide hydrogen bonding (black dashes) and intersheet interactions (Lys–Ser and Lys–Thr, blue dashes; Lys–C terminus, red dashes). Graphics generated using PyMol.^12^

Hydrogen bonding is the most directional and strongest of the interactions present, promoting one‐dimensional growth along the *a* dimension—consistent with the tape morphology (Figure 1) and architecture (Figure 2). Intersheet side chain interactions stabilize the broadening of the tape including Lys–Ser/Lys–Thr hydrogen bonding, Lys–C terminus interactions and aromatic Tyr interactions. Finally, the amphiphilic nature of the peptide arrangement encourages the formation of a stable bilayer defining the thickness of the tape. As a result of this arrangement, the edge of the tape will expose the hydrophobic *bc* plane (Figure 3 a) to the solvent, which could finally be buried by nanotube formation. The structure strikes a balance between self‐assembly and bending of the formed bilayer resulting in a controlled tube diameter (Figure 2 a,b).

The final model structure was validated by comparison of calculated to experimental diffraction data. The major reflections at positions 29, 9.8 and 4.8 Å pertaining to the major structural features of the model are accurately reproduced in the positions of the calculated reflections as shown in Figure 4 (see also Figure S7). Some additional information is observed in the calculated patterns but this may be due to a number of factors: discrepancies in crystallite size and packing, disorder, side chain and backbone conformation for end amino acids, and the simulation of perfect crystallinity. The relative intensities of reflections will be modulated by the exact structural architecture and side chain rotamers within the unit cell and this is difficult to exactly reproduce in this model. However, it is interesting that in this structural arrangement, stabilizing side chain interactions between peptides that rationalize the formation and stabilization of the nanotubes are also found. Further, the ability to observe excellent matching between calculated and experimental diffraction data for the film texture, with the parallel and perpendicular beam orientations reinforces the validity of the model. Taken together, the match between calculated and experimental diffraction data indicates that the unit cell and model structure are representative of the tapes that constitute the αSβ1 nanotube wall.

**Figure 4:**
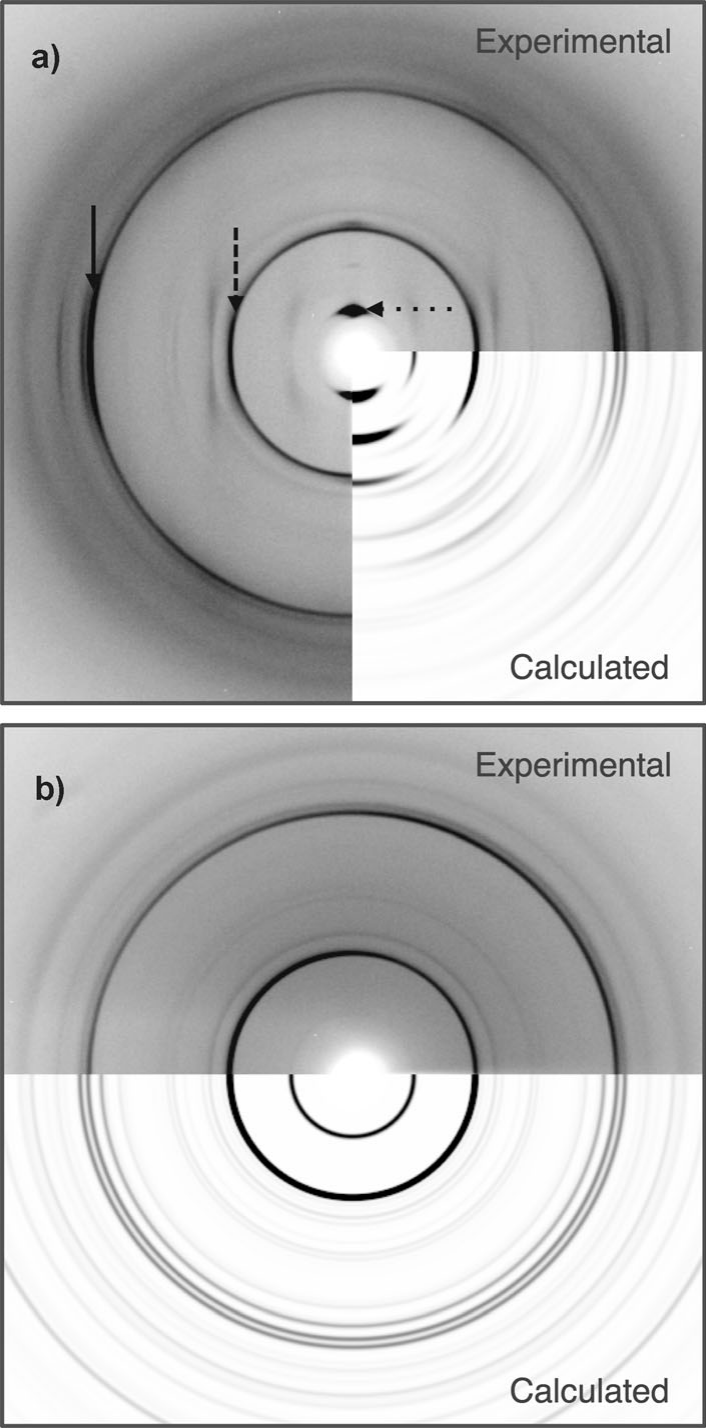
Accurate reproduction of the experimental X‐ray fiber diffraction data collected from αSβ1 peptide assemblies by diffraction calculation from the proposed model structure in the film texture. a) Comparison of film‐textured XRFD patterns of αSβ1 is made to calculated patterns with the beam parallel and, b) perpendicular to the film plane. Reflections are highlighted at 4.8 Å (solid arrow), 9.8 Å (dashed arrow), and 29 Å (dotted arrow).

The cross‐β structure is clearly present in this system but the texture observed in the XRFD patterns and modeled interactions shown here describe the unusual orientation and molecular arrangement of these giving rise to a nanotubular morphology. Helical tapes and nanotubes have been observed for a number of peptidic self‐assembling monomers including Aβ(16‐22),10a,b, 13 cyclo[(‐d‐Ala‐l‐Glu‐d‐Ala‐l‐Gln‐)2],6c Lanreotide,6a A6 K,10d NDI‐lysine amphiphiles,6b KLVFF‐derived peptides,^9^, 10c and FFFEEE‐containing peptide amphiphiles.^14^

In summary, the αSβ1 peptide assembles into flat tape structures with a repetitive separation of 4.8 Å along the tape long axis. These tapes consist of a peptide bilayer structure, which can be modeled based on the cross‐β structure found in amyloid proteins. These tapes assemble over relatively large widths greater than 300 nm and of indeterminate lengths. They are stabilized by hydrogen bonding along their tape long axes, peptide packing and side chain interactions stabilize the lateral tape width, whilst the amphiphilic nature of the peptide results in the thin bilayer structure. To further stabilize the structure, these tapes may then twist to form helical tapes, which subsequently close into nanotubes of ca. 240 nm as observed here. As described earlier, the difference in tube diameter to other nanotube‐forming peptide systems is striking and we speculate that it is related to intrinsic β‐sheet twist properties related to the sequence of self‐assembling peptide but also the sequence‐dependent free‐energy cost of tape bending versus minimization of hydrophobic exposure. The orientation of this molecular arrangement within the nanotube wall creates a cross‐β bilayer architecture similar to those formed by other unrelated peptide sequences,10b, 13, 15 but unique in its parallel amphiphilic nature. The molecular structure presented here provides an explanation of the elaborate morphology of these nanotubes and may also have relevance to the underlying architecture of related nanotubes formed by other peptides.

## Supplementary Material

As a service to our authors and readers, this journal provides supporting information supplied by the authors. Such materials are peer reviewed and may be re‐organized for online delivery, but are not copy‐edited or typeset. Technical support issues arising from supporting information (other than missing files) should be addressed to the authors.

miscellaneous_information

## References

[1a] F. Chiti, C. M. Dobson, Annu. Rev. Biochem. 2006, 75, 333–366; 16756495 10.1146/annurev.biochem.75.101304.123901

[1b] P. Sikorski, E. D. T. Atkins, L. C. Serpell, Structure 2003, 11, 915–926; 12906823 10.1016/s0969-2126(03)00149-7

[1c] T. R. Jahn, O. S. Makin, K. L. Morris, K. E. Marshall, P. Tian, P. Sikorski, L. C. Serpell, J. Mol. Biol. 2010, 395, 717–727. 19781557 10.1016/j.jmb.2009.09.039

[2a] K. Morris, L. Serpell, Chem. Soc. Rev. 2010, 39, 3445–3453; 20668734 10.1039/b919453n

[2b] I. W. Hamley, Angew. Chem. 2007, 119, 8274–8295;

[2c] J. B. Matson, R. H. Zha, S. I. Stupp, Curr. Opin. Solid State Mater. Sci. 2011, 15, 225–235. 22125413 10.1016/j.cossms.2011.08.001 PMC3224089

[3] M. Goedert, Nat. Rev. Neurosci. 2001, 2, 492–501. 11433374 10.1038/35081564

[4a] A. J. Geddes, K. D. Parker, E. D. Atkins, E. Beighton, J. Mol. Biol. 1968, 32, 343–358; 5643439 10.1016/0022-2836(68)90014-4

[4b] E. D. Eanes, G. G. Glenner, J. Histochem. Cytochem. 1968, 16, 673–677; 5723775 10.1177/16.11.673

[4c] L. C. Serpell, Biochim. Biophys. Acta Mol. Basis Dis. 2000, 1502, 16–30. 10.1016/s0925-4439(00)00029-6 10899428

[5a] U. Slotta, S. Hess, K. Spiess, T. Stromer, L. Serpell, T. Scheibel, Macromol. Biosci. 2007, 7, 183–188; 17295405 10.1002/mabi.200600201

[5b] M. Heim, L. Roemer, T. Scheibel, Chem. Soc. Rev. 2010, 39, 156–164. 20023846 10.1039/b813273a

[6a] C. Valery, M. Paternostre, B. Robert, T. Gulik‐Krzywicki, T. Narayanan, J. C. Dedieu, G. Keller, M. L. Torres, R. Cherif‐Cheikh, P. Calvo, F. Artzner, Proc. Natl. Acad. Sci. USA 2003, 100, 10258–10262; 12930900 10.1073/pnas.1730609100 PMC193548

[6b] H. Shao, M. Gao, S. H. Kim, C. P. Jaroniec, J. R. Parquette, Chem. Eur. J. 2011, 17, 12882–12885; 22167876 10.1002/chem.201102616

[6c] M. R. Ghadiri, J. R. Granja, R. A. Milligan, D. E. McRee, N. Khazanovich, Nature 1993, 366, 324–327. 8247126 10.1038/366324a0

[7] L. C. Serpell, J. Berriman, R. Jakes, M. Goedert, R. A. Crowther, Proc. Natl. Acad. Sci. USA 2000, 97, 4897–4902. 10781096 10.1073/pnas.97.9.4897 PMC18329

[8a] H. Heise, W. Hoyer, S. Becker, O. C. Andronesi, D. Riedel, M. Baldus, Proc. Natl. Acad. Sci. USA 2005, 102, 15871–15876; 16247008 10.1073/pnas.0506109102 PMC1276071

[8b] M. Vilar, H. T. Chou, T. Luhrs, S. K. Maji, D. Riek‐Loher, R. Verel, G. Manning, H. Stahlberg, R. Riek, Proc. Natl. Acad. Sci. USA 2008, 105, 8637–8642; 18550842 10.1073/pnas.0712179105 PMC2438424

[8c] H. Heise, M. S. Celej, S. Becker, D. Riede, A. Pelah, A. Kumar, T. M. Jovin, M. Baldus, J. Mol. Biol. 2008, 380, 444–450; 18539297 10.1016/j.jmb.2008.05.026

[8d] G. Comellas, L. R. Lemkau, A. J. Nieuwkoop, K. D. Kloepper, D. T. Ladror, R. Ebisu, W. S. Woods, A. S. Lipton, J. M. George, C. M. Rienstra, J. Mol. Biol. 2011, 411, 881–895. 21718702 10.1016/j.jmb.2011.06.026 PMC3157309

[9] J. Adamcik, V. Castelletto, S. Bolisetty, I. W. Hamley, R. Mezzenga, Angew. Chem. 2011, 123, 5609–5612; 10.1002/anie.201100807 21538748

[10a] K. Lu, J. Jacob, P. Thiyagarajan, V. P. Conticello, D. G. Lynn, J. Am. Chem. Soc. 2003, 125, 6391–6393; 12785778 10.1021/ja0341642

[10b] W. S. Childers, A. K. Mehta, R. Ni, J. V. Taylor, D. G. Lynn, Angew. Chem. 2010, 122, 4198–4201;

[10c] V. Castelletto, I. W. Hamley, P. J. F. Harris, U. Olsson, N. Spencer, J. Phys. Chem. B 2009, 113, 9978–9987; 19555054 10.1021/jp902860a

[10d] S. Bucak, C. Cenker, I. Nasir, U. Olsson, M. Zackrisson, Langmuir 2009, 25, 4262–4265. 19275132 10.1021/la804175h

[11] S. H. White, W. C. Wimley, Annu. Rev. Biophys. Biomol. Struct. 1999, 28, 319–365. 10410805 10.1146/annurev.biophys.28.1.319

[12] The PyMOL Molecular Graphics System, Version 0.99rc6, Schrödinger, LLC.

[13] A. K. Mehta, K. Lu, W. S. Childers, Y. Liang, S. N. Dublin, J. Dong, J. P. Snyder, S. V. Pingali, P. Thiyagarajan, D. G. Lynn, J. Am. Chem. Soc. 2008, 130, 9829–9835. 18593163 10.1021/ja801511n

[14] E. T. Pashuck, S. I. Stupp, J. Am. Chem. Soc. 2010, 132, 8819–8821. 20552966 10.1021/ja100613w PMC3116515

[15] J. Madine, H. A. Davies, C. Shaw, I. W. Hamley, D. A. Middleton, Chem. Commun. 2012, 48, 2976–2978. 10.1039/c2cc17118j 22328992

