# Supplementary material for: The Structure of Cross‐β Tapes and Tubes Formed by an Octapeptide, αSβ1
Source: Angew Chem Int Ed Engl. 2013 Jan 10;52(8):2279–83. doi: 10.1002/anie.201207699 (PMC4279883; doi:10.1002/anie.201207699)
Supplement: Supplementary file 1 — miscellaneous_information [file anie-52-2279-s1.pdf]

Angewandte Chemie
Eine Zeitschrift der Gesellschaft Deutscher Chemiker

Supporting Information

© Wiley-VCH 2013

69451 Weinheim, Germany

The Structure of Cross-β Tapes and Tubes Formed by an Octapeptide, αSβ1**

*Kyle L. Morris, Shahin Zibaee, Lin Chen, Michel Goedert, Pawel Sikorski, and Louise C. Serpell**

anie_201207699_sm_miscellaneous_information.pdf

SUPPORTING INFORMATION

Experimental Section

Self-assembly conditions. A peptide with the sequence NH_2-VLYVGSKT-COOH was prepared by Fmoc chemistry on Wang resin, purity = 95% (Advanced Biomedical Ltd, Oldham, UK). The peptide was initially completely dissolved in 0.2 µm filtered milliQ distilled water (18.2 MO) at approximately 15 $mgmL^{-1}$. In order to increase the peptide concentration, the solvent was reduced by centrifugal evaporation (Savant SpeedVac) at 40 – 45 °C for a total of 90 minutes. Once the peptide solution was reduced by as much as 10-fold, a homogenous opaque white gel appeared. Based on the reduction in solution volume, the concentration was estimated to be ~150 – 200 $mgmL^{-1}$.

Transmission electron microscopy. Samples were prepared by allowing 4 µL of solution to adsorb to a TEM Formvar-carbon film coated 400-mesh copper grid (Agar scientific) surface for one minute and excess was blotted with grade I filter paper (Whatman). Grids were washed by one-minute incubation with 0.2 µm filtered milliQ water (18.2 MO), excess blotted, and subsequently stained and blotted by two, one minute incubations with 2% w/v uranyl acetate (Sigma-Aldrich). Grids were inspected using a Hitachi 7100 TEM (Hitachi) operated at 100 kV. Images were acquired digitally using an axially mounted Gatan Ultrascan 1000 CCD camera (Gatan, Oxford, UK). Upper and lower frequency data was filtered using the fast Fourier transform bandpass filters implemented in ImageJ (NIH) for clear morphology visualisation.[1]

X-ray fibre diffraction. Fibres were aligned by a number of methods to create different sample textures (fibrous and film) such that the maximal amount of information could be obtained.[2] For the fibrous texture, a 10 µL droplet of fibril solution was suspended between two wax-tipped 1.2 mm O.D, 0.94 mm I.D borosilicate capillaries (Harvard apparatus) and placed in a parafilm-sealed petri dish at room temperature. The suspension was allowed to dry by evaporation. For the film texture, a 50 µL droplet of fibril solution was aspirated into a X-ray transmissible 0.7 mm borosilicate capillary (Capillary Tube Suppliers Ltd) and sealed at one end to prevent further capillary action. The capillary was allowed to dry by evaporation to create a film texture. Aligned specimens were mounted and aligned on a goniometer head. For the fibrous texture, the beam axis was carefully aligned perpendicular to the fibre axis. Exposures were taken at F 0 and 90° (?F 0.5) about the fibre axis to ensure cylindrical averaging. For film-textured alignments, samples were mounted and aligned on the goniometer head ensuring the beam axis was parallel to the plane of the film. Exposures were taken at F 0 and 90° (?F 0.5) resulting in diffraction patterns with the beam axis parallel and perpendicular to the film plane. Diffraction data were collected using a Rigaku 007HF Cu Ka (? 1.5419 Å) rotating anode generator with VariMax-HF mirrors and Saturn 944+ CCD detector. Exposure times were typically 10 – 120 seconds and specimen to detector distances 50 or 100 mm.

The detector recorded images at 1042 x 1042 pixels in (2 x 2 binning mode) at 90 $µmpixel^{-1}$, whilst images for processing were compressed to 523 x 523 pixels at 179.3 $µmpixel^{-1}$ using Mosflm 7.0.5[3] and subsequently saved in .tiff format. Fibre diffraction patterns were processed and analysed in Clearer.[4] Signal positions were determined by Clearer. Any signals measured manually are indicated so by the use of an asterix (*). Azimuthal reflection angles were calculated in Matlab (Mathworks Inc) and intensities integrated over 4 pixels. These data were processed by a Savitzky-Golay filter[5] and azimuthal reflection angles reported from the centre of the reflection peaks.

Model building and refinement. The final model construction of the molecular architecture of aSß1 was informed by iterative model building where we explored various parallel and antiparallel architectures. Models of aSß1 were built using Chimera in an idealised geometries and subsequently minimized within the same package.[6] Briefly, using the unit cell dimensions a [3 3 3] lattice was created to prevent edge effects in the minimisation. Hydrogen atoms were added and charges assigned using the integrated MMTK[7] and the AMBER ff99SB forcefield.[8] Steepest decent and conjugate gradient minimisation methods were used over 200 and 100 steps respectively at a step size of 0.02 Å. Minimisation was iteratively performed by initial rigid body and subsequent backbone refinement. Model quality was quantitatively assessed by MolProbity analysis.[9] Model coordinates were recorded in PDB format.

Diffraction pattern calculation. Fibre diffraction patterns were calculated from newly built PDB models using the Fibre Diffraction Simulation module of Clearer.[4] Diffraction settings were input to match the experimental pattern. To simulate the film texture, the rotation axis (assigned Fibre Axis in Clearer) was set as (0, 0, 1), with a crystallite size of 80 x 80 x 50 nm and unit cell dimensions of a = 9.50 Å; b = 19.92 Å; c = 27.97 Å; a = ß = ? = 90.0°. The fibre disorder parameters $s_?$ and $s_?$ were infinite and 0.25 radians respectively with a sampling interval of 1 pixel. Contrast was changed according to the best visualisation of the pattern signals. Detector size was adjusted to the size of the experimental .tiff pattern. All other parameters were set to default values.[4]

Figures and Tables

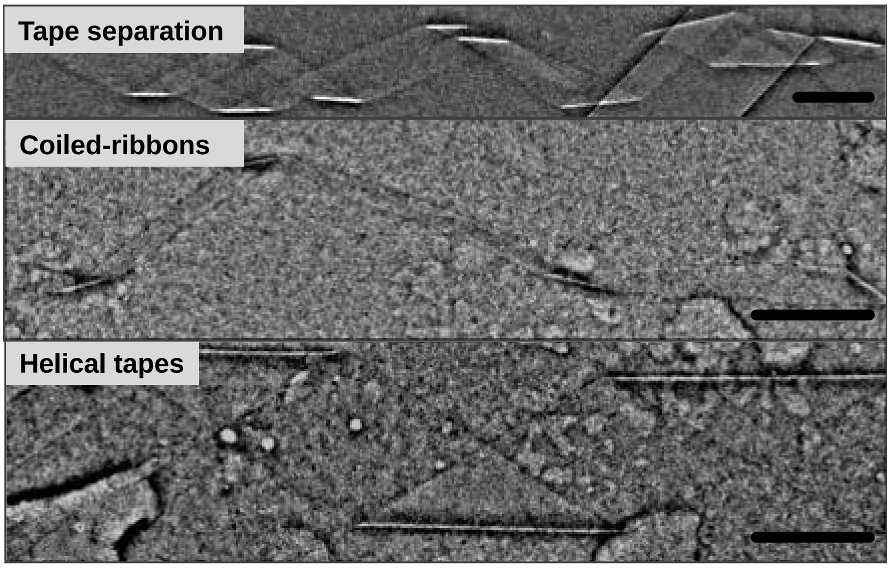

Figure S1. TEM showing the variable morphologies formed by aSß1. The morphology may be described as tapes, coiled ribbons and helical tapes.[10] Micrographs were processed with ImageJ and using fast Fourier transform bandpass filtering.[1] Black scale bars correspond to 200 nm.

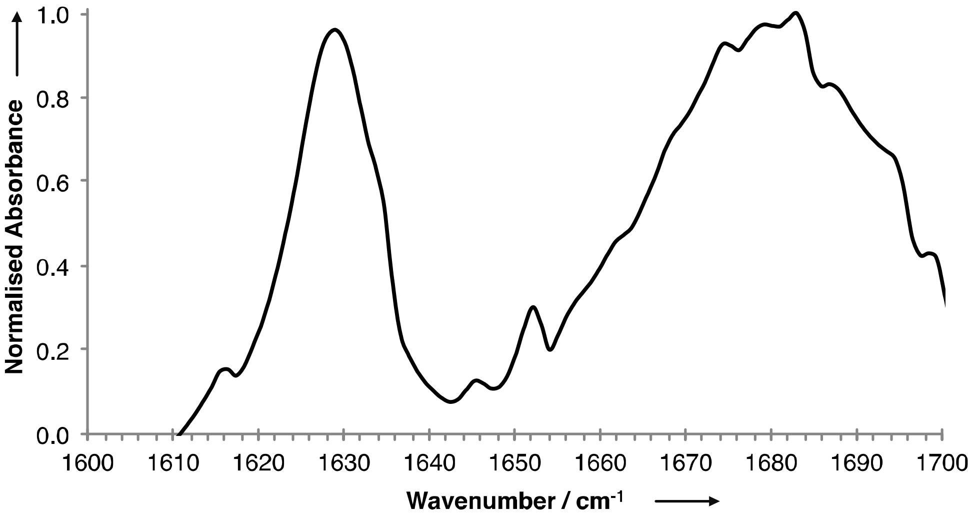

Figure S2. FTIR of aSß1 assemblies. The spectrum indicates predominantly ß-sheet secondary structure. Spectra were collected on Bruker FTIR at 2 cm^{-1} resolution by averaging over 128 scans. The aSß1 sample was loaded onto a CaF_2 window, and another CaF_2 window was placed on the top of sample. The space between two windows was not controlled. The spectrum was background and H_2O subtracted. FTIR spectra over the range from 1600 to 1700 cm^{-1} where the characteristic amide I band appears were obtained to determine secondary structure.

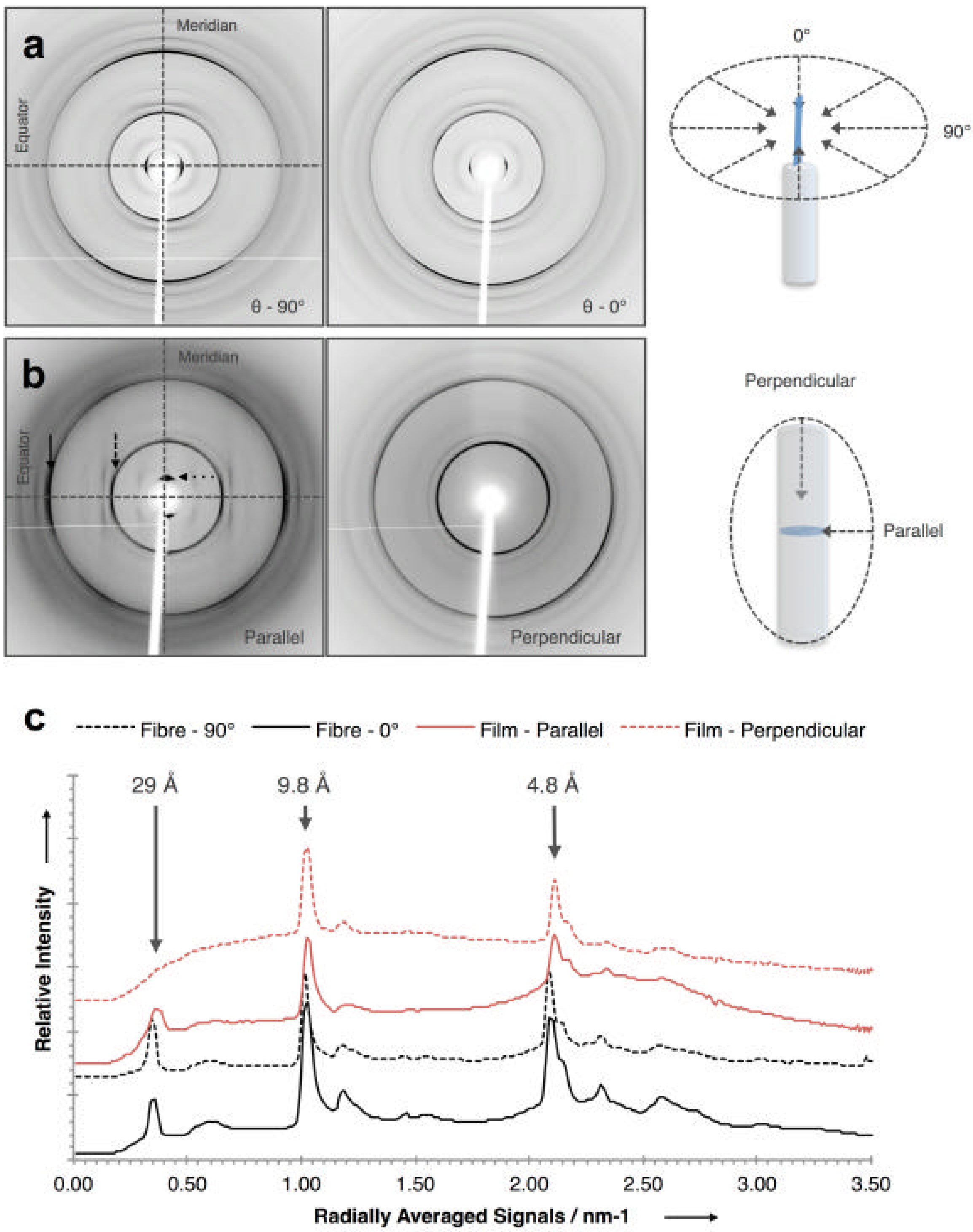

Figure S3. X-ray fibre diffraction patterns collected from fibrous and film textured of alignments of fibrils of aSß1. a) Diffraction patterns collected from a fibrous texture and b) film texture sample. Reflections are highlighted at 4.7 – 8 Å (solid arrow), 9.8 Å (dashed arrow) and 29 Å (dotted arrow). Schematics of the alignments are shown with the X-ray beam incidence shown as dotted arrows. Parallel and perpendicular terminology refers to the beam position relative to the plane of the film. c) The diffraction signals exhibited by the two textures of aSß1 are radially averaged and compared graphically. The reflections are comparable between patterns indicating that the interatomic spacings in each textured alignment are equivalent. The diffraction signals expected for a cross-ß structure are observed in the fibrous alignment at 4.7 and 9.8 Å. The fibrous 9.8 Å reflection usually observed on the equator,[2] arising from the separation of ß-sheets, is found on the meridian in the fibrous pattern (a). On the film-textured pattern (b) both the 4.8 Å and 9.8 Å reflections are equatorial and a strong 29 Å meridional reflection is observed. In this texture, the pattern is rotated compared to the fibrous texture such that that meridional axis (the molecular rotation axis) is perpendicular to the plane of the film, indicating that the 29 Å spacing is aligned perpendicular to the film plane. There is also a low intensity meridional reflection at 9.4 Å, also close to the common cross-ß intersheet distance of approximately 10 Å. A pattern collected from the film with beam axis perpendicular to the film plane shows the expected rings arising from the sample containing anisotropic structures orientated about the plane of the film but 29 Å signal is not observed (b). These observations are consistent with the tape structures observed in the TEM aligning with their long axes parallel to the film plane (Fig. S5a) and indicate a novel orientation of the aSß1 molecules within these assemblies.

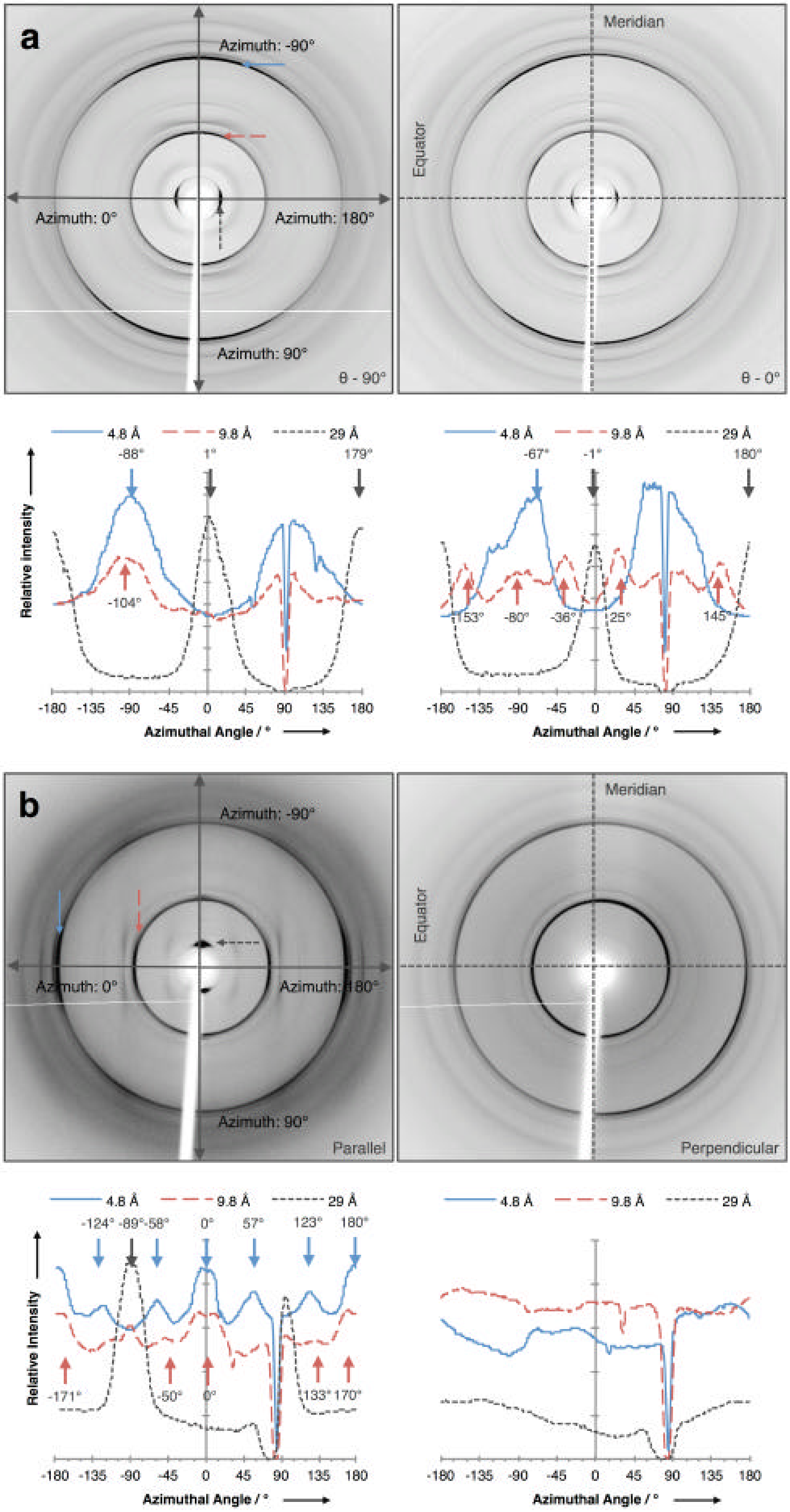

a
Azimuth: -90°
Azimuth: 0°
Azimuth: 180°
Azimuth: 90°
θ - 90°
Meridian
Equator
θ - 0°
4.8 Å
9.8 Å
29 Å
-88°
1°
179°
-104°
Relative intensity
Azimuthal Angle / °
-67°
-1°
180°
-153°
-80°
-36°
25°
145°
b
Azimuth: -90°
Azimuth: 0°
Azimuth: 180°
Azimuth: 90°
Parallel
Meridian
Equator
Perpendicular
-124°
-89°
-58°
0°
57°
123°
180°
-171°
-50°
0°
133°
170°
Relative Intensity
Azimuthal Angle / °

Figure S4. Azimuthal integration of the major reflections of the X-ray fibre diffraction patterns in (a) the fibre texture and (b) the film texture. Blue – 4.8 Å, Red long dash – 9.8 Å, Black short dash – 29 Å. The approximately -89° alignment of the 9.8 Å reflection in (b) is a result of the overlapping 9.4 Å meridional reflection. The beam stop occludes reflection measurement at ~90° in each of the patterns.

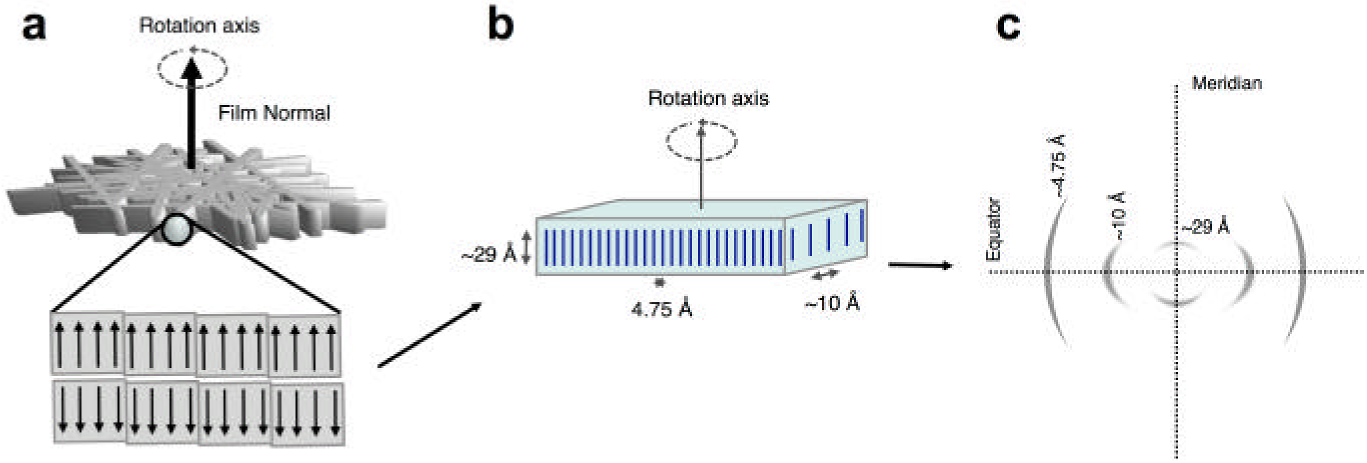

Figure S5. The arrangement of crystallites within the film-textured alignment of aSß1. a) The film comprises of flat tapes with perpendicularly arranged ß-strands. b) The determined unit cell orientation within these is shown with c) the expected idealized X-ray fibre diffraction pattern from this lattice arrangement.

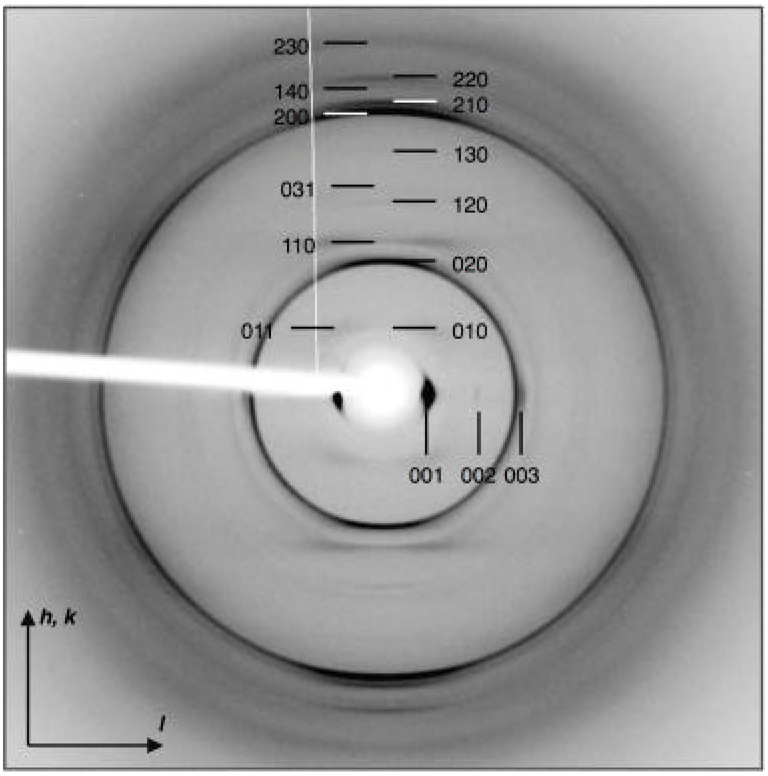

Figure S6. Indexing of the film-textured X-ray fibre diffraction pattern. aSß1 assembles in a cell of the dimensions *a* = 9.50; *b* = 19.92; *c* = 27.97; a = ß = ? = 90.0°, indexing of reflections is shown in Table S2.

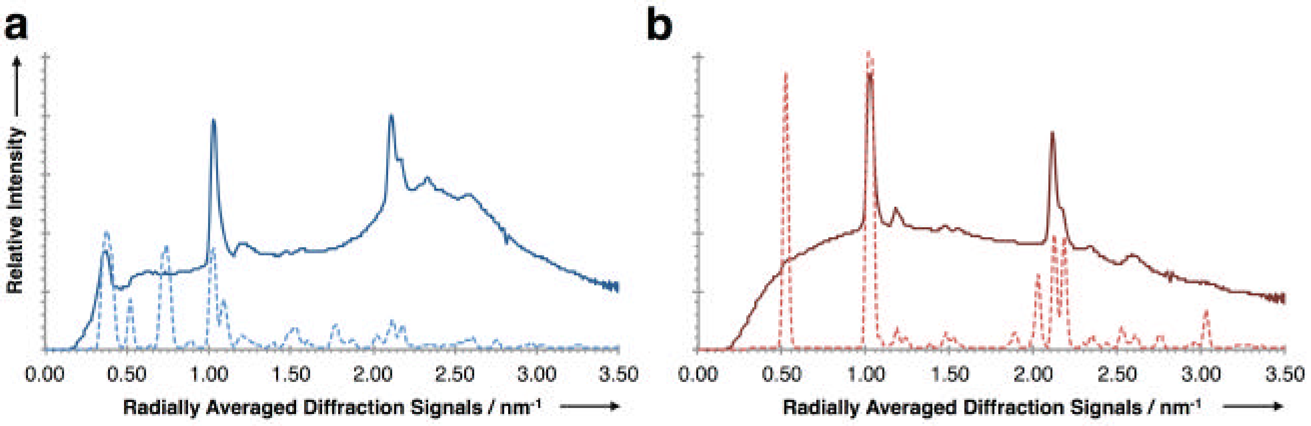

Figure S7. Comparison of the experimental and calculated X-ray fibre diffraction from aSß1. In both cases with a) the beam parallel and b) perpendicular to the film plane, the calculated patterns accurately reproduce the experimental patterns as evident in the radially averaged graphical traces. Solid lines – experimental X-ray fibre diffraction data, dashed lines – calculated data.

Table S1. Table of X-ray fibre diffraction reflections from fibrous- and film-textured alignments of aSß1. M – Meridional, E- Equatorial, O – Off-axis, Rad – Radially averaged, * measured manually.

Fibrous – 90°			Fibrous – 0°		
d-Spacings (Å)	Normalized Intensity	Axis	d-Spacings (Å)	Normalized Intensity	Axis
29.6	0.89	E	30.0	0.81	E
16.6	0.12	O	16.8	0.26	O
9.95	0.86	M	9.94	1.00	O
8.58	0.31	M	8.58	0.46	M
6.93	0.15	M	6.95	0.26	M
6.56	0.12	Rad	6.58	0.21	Rad
5.44	0.11	M	5.45	0.21	M
4.81	1.00	M	4.79	0.96	M
4.69	0.47	M	4.69	0.76	M
4.46	0.23	Rad	-	-	-
4.34	0.34	M	4.34	0.52	M
3.90	0.21	M	3.90	0.38	Rad
3.70	0.15	Rad	-	-	-
-	-	-	3.33	0.20	Rad
-	-	-	3.16	0.17	Rad
3.08	0.08	M	-	-	-

Film – Parallel			Film – Perpendicular		
d-Spacings (Å)	Normalized Intensity	Axis	d-Spacings (Å)	Normalized Intensity	Axis
28.6	0.48	M	-	-	-
-	-	-	19.6	*	Rad
17.0	0.31	O	-	-	-
13.9	*	M	-	-	-
9.82	1.00	E	9.86	1.00	Rad
9.44	*	M	-	-	-
8.62	*	E	8.53	0.51	Rad
6.85	0.39	E	6.86	0.45	Rad
6.47	0.39	E	6.53	0.44	Rad
5.44	*	E	5.45	0.40	Rad
4.75	0.96	E	4.75	0.79	Rad
4.65	0.79	E	4.64	*	Rad
4.41	*	E	4.39	0.37	Rad
4.30	0.67	E	4.31	0.38	Rad
3.88	0.62	E	3.88	0.34	Rad

Table S2. Unit cell indexing of the X-ray fibre diffraction signals exhibited by assembled aSß1 in the film-texture.

Dimensions	*a* (Å)	*b* (Å)	*c* (Å)	a = ß = ? (°)
	9.50	19.92	27.97	90.00
Observed / Å	*h*	*k*	*l*	Calculated / Å
28.6	0	0	1	28.0
19.6	0	1	0	19.9
17.0	0	1	1	16.2
13.9	0	0	2	14.0
9.82	0	2	0	9.96
9.44	0	0	3	9.32
8.62	1	1	0	8.58
6.85	1	2	0	6.87
6.47	0	3	1	6.46
5.44	1	3	0	5.44
4.75	2	0	0	4.75
4.65	2	1	0	4.62
4.41	1	4	0	4.41
4.30	2	2	0	4.29
3.88	2	3	0	3.86

References

[1] A. M. Abramoff, P. J. Magelhaes, S. J. Ram, *Biophotonics International* **2004**, *11*, 36-42.
[2] K. L. Morris, L. C. Serpell, in *Amyloid Proteins: Methods and Protocols, Second Edition, Vol. 849* (Eds.: E. M. Sigurdsson, M. Calero, M. Gasset), Springer, **2012**, pp. 121-135.
[3] A. G. W. Leslie, in *Joint CCP4 + ESF-EAMCB Newsletter on Protein Crystallography, Vol. No. 26.*, No. 26. ed., **1992**.
[4] O. S. Makin, P. Sikorski, L. C. Serpell, *J. Appl. Crystallogr.* **2007**, *40*, 966-972.
[5] A. Savitzky, M. J. E. Golay, *Anal. Chem.* **1964**, *36*, 1627-1639.
[6] E. F. Pettersen, T. D. Goddard, C. C. Huang, G. S. Couch, D. M. Greenblatt, E. C. Meng, T. E. Ferrin, *J. Comput. Chem.* **2004**, *25*, 1605-1612.
[7] K. Hinsen, *J. Comput. Chem.* **2000**, *21*, 79-85.
[8] J. M. Wang, P. Cieplak, P. A. Kollman, *J. Comput. Chem.* **2000**, *21*, 1049-1074.
[9] I. W. Davis, L. W. Murray, J. S. Richardson, D. C. Richardson, *Nucleic Acids Res.* **2004**, *32*, 615-619.
[10] H. Shao, M. Gao, S. H. Kim, C. P. Jaroniec, J. R. Parquette, *Chemistry-a European Journal* **2011**, *17*, 12882-12885.
